# Environmental Hypertonicity Causes Induction of Gluconeogenesis in the Air-Breathing Singhi Catfish, *Heteropneustes fossilis*

**DOI:** 10.1371/journal.pone.0085535

**Published:** 2013-12-20

**Authors:** Manas Das, Bodhisattwa Banerjee, Mahua G. Choudhury, Nirmalendu Saha

**Affiliations:** Biochemical Adaptation Laboratory, Department of Zoology, North-Eastern Hill University, Shillong, India; National University of Singapore, Singapore

## Abstract

The air-breathing singhi catfish (*Heteropneustes fossilis*) is frequently being challenged by different environmental insults such as hyper-ammonia, dehydration and osmotic stresses in their natural habitats throughout the year. The present study investigated the effect of hyperosmotic stress, due to exposure to hypertonic environment (300 mM mannitol) for 14 days, on gluconeogenesis in this catfish. *In situ* exposure to hypertonic environment led to significant stimulation of gluconeogenic fluxes from the perfused liver after 7 days of exposure, followed by further increase after 14 days in presence of three different potential gluconeogenic substrates (lactate, pyruvate and glutamate). Environmental hypertonicity also caused a significant increase of activities of key gluconeogenic enzymes, namely phosphoenolpyruvate carboxykinase, fructose 1, 6-bisphosphatase and glucose 6-phosphatase by about 2-6 fold in liver, and 3-6 fold in kidney tissues. This was accompanied by more abundance of enzyme proteins by about 1.8–3.7 fold and mRNAs by about 2.2–5.2 fold in both the tissues with a maximum increase after 14 days of exposure. Hence, the increase in activities of key gluconeogenic enzymes under hypertonic stress appeared to be as a result of transcriptional regulation of genes. Immunocytochemical analysis further confirmed the tissue specific localized expression of these enzymes in both the tissues with the possibility of expressing more in the same localized places. The induction of gluconeogenesis during exposure to environmental hypertonicity possibly occurs as a consequence of changes in hydration status/cell volume of different cell types. Thus, these adaptational strategies related to gluconeogenesis that are observed in this catfish under hypertonic stress probably help in maintaining glucose homeostasis and also for a proper energy supply to support metabolic demands mainly for ion transport and other altered metabolic processes under various environmental hypertonic stress-related insults.

## Introduction

Gluconeogenesis from lactate, pyruvate and amino acids is important for the maintenance of circulating glucose level during strenuous [1] and fasting conditions in vertebrates [2]. Gluconeogenesis has been extensively studied in liver and kidney tissues of various fish species, since these two organs are the major sites of this metabolic pathway [3-5]. In some teleostean fish, gluconeogenesis occurs at relatively higher rates [6-10], and is thought to be a key process in maintaining glucose homeostasis [11], especially in carnivorous fish that have high protein and low carbohydrate diets [12]. Further, carbohydrate may also be used for short term responses in acute stress situations as a last resort in fish [13]. Even though most of the enzymes involved in glucose metabolism have been detected in fish, the regulation of carbohydrate metabolism differs in some aspects from that of mammals [14]. The regulation of hepatic glucose metabolism in teleost fish is reported to be influenced by different stressful conditions, such as low dietary carbohydrates and changes in hepatocellular hydration status [15-17].

Cells respond to changes in osmotic pressure with compensatory molecular adaptations that allow them to reestablish homeostasis of osmotically disturbed aspects of cell structure and function [18]. A remarkable property of living cells is their ability to maintain a comparatively constant cell volume under different physiological conditions (for reviews, see 19,20). Thus, cells restore their conserved ionic milieu, chiefly by adjusting the levels of compatible osmolytes [21]. Cell volume may be challenged by a variety of factors such as the intestinal absorption of water, and of various amino acids and metabolites, or by exposure to different osmotic environments especially in the case of aquatic animals. Most cells possess various volume-regulatory mechanisms such as regulatory volume decrease (RVD) and regulatory volume increase (RVI) to maintain the constancy of cell volume and also the hydration status of the cell largely by changing the permeability of various ions such as K^+^, Na^+^, H^+^, Cl^–^ and HCO_3_^-^, and certain organic osmolytes [19,22-24]. However, it has been noticed in many cell types that they remain either in a slightly swollen or shrunken state for the duration of the anisotonic exposure (for review, see 19). Irrespective of the route of RVD or RVI, increase in hepatic cell volume generally results in increased anabolism and curtailment of catabolic pathways, while the reverse is true during the decrease in hepatic cell volume [16,25-28].

More recently, it has been demonstrated that the liver cells of the air-breathing walking catfish (*Clarias batrachus*) possess efficient volume regulatory mechanisms, but remain in partly swollen or shrunken state as long as they are exposed to anisotonicity [23]. These changes of cell volume due to anisotonicity have been reported to cause changes in glucose, pyruvate and lactate fluxes, glycogen metabolism [16], hexose monophosphate pathway [29], and also on gluconeogenesis [17] in the perfused liver of walking catfish. Hallgren et al. [30] also reported similar effects of cell volume changes at least on glycogen metabolism in the hepatocytes of three fish species. However, it has been noticed that teleost fish face more problems of osmotic stress in comparison with mammals primarily owing to osmolarity changes in their external environment. The air-breathing singhi catfish (*Heteropneustes fossilis*), found predominantly in tropical Southeast Asia, is reported to be more resistant to various environmental challenges such as high environmental ammonia, hypoxic and desiccation stresses (for reviews, see 31,32). Further, they are reported to be euryhaline, inhabiting fresh and brackish waters as well as muddy marshes, thus facing wide variations of external osmolarity changes ranging from 100-350 mOsmol.l^-1^ [33]. They frequently encounter the problem of osmolarity changes in the same habitat during different seasons of the year, especially in summer when the ponds and lakes dry up, thus compelling them to migrate inside the mud peat to avoid total dehydration, and during the monsoon season when the water in the same habitat gets diluted. Thus, looking at its enormous capacity in challenging the external osmolarity changes, the present study was aimed at in elucidating the possible effect of environmental hypertonicity on gluconeogenesis in this singhi catfish.

## Materials and Methods

### Animal

The air-breathing singhi catfish (*Heteropneustes fossilis*) weighing 60 ± 10 g body mass were purchased from a single source that are bred and cultured in selected commercial ponds. Fishes were acclimatized in the laboratory approximately for 1 month at 28 ± 2 °C with 12 h:12 h light and dark photoperiods before experiments. No sex differentiation of the fish was done while performing these studies. Minced dry fish and rice bran (5% of body wt) were given as food every day, and the water, collected from a natural stream, was changed on alternate days. Experiments were conducted after one month of acclimatization when the food consumption became normal and mortality rate became zero. Food was withdrawn 24 h prior to experiments.

### Ethics Statement

Fishes were purchased from single source that are bred and cultured in selected commercial ponds in Lumding situated in the state of Assam, India. Fishes were anaesthetized in neutralized 3-aminobenzoic acid ethyl ester (MS-222, 0.2 g.l^-1^) before sacrificing by decapitation. The study was approved by the Institutional Animal Ethics Committee (IAEC) of North-Eastern Hill University, Shillong, India.

### Experimental set up

Two groups of fish of similar sizes having five fish in each group were placed in two plastic containers having 5 L each of 300 mM mannitol (equivalent to water osmolarity of 300 mOsmo.l^-1^l) solution prepared in bacteria-free filtered stream water (pH 7.15 ± 0.07). Another two groups of fish were kept in two plastic containers having 5 L each of bacteria-free filtered stream water (pH 7.05 ± 0.04) and served as controls. Solutions from each bucket were replaced with fresh media every day at a fixed time. After 7 and 14 days, five fish each from control and treated containers were anaesthetized in neutralized 3-aminobenzoic acid ethyl ester (MS-222, 0.2 g.l^-1^) for 5 min. Blood samples were collected from the caudal vasculature with a heparinized syringe, and liver and kidney tissues were dissected out, plunged into liquid nitrogen and stored at −80 °C. All analyses in different tissues were completed within 2-3 weeks of collecting tissues.

Another set of treated and control fish were used for perfusion experiments after 7 and 14 days of experimental set-up.

### Blood sampling and osmolarity measurement

The blood was collected with a heparinized syringe from the caudal vein and centrifuged at 10,000×g for 10 min at 0 ± 2 °C for separating out the plasma from blood leucocytes. The plasma osmolarity was measured with a Camlab osmometer (Model 200) using the freezing point depression method.

### Measurement of water content

The water content in cells of different tissues of both control and hypertonically-treated fish was determined by oven drying method following Goswami and Saha [16].

### Liver perfusion technique

Fishes were anaesthetized in neutralized 3-aminobenzoic acid ethyl ester (MS-222, 0.2 g/l) for 5 min before operation to perform the liver perfusion. The livers, while remaining attached to the body, were perfused via the portal vein in a non-circulating manner with haemoglobin-free medium following the method described by Saha et al. [34]. The isotonic medium (265 mOsmol.l^-1^, determined by freezing point depression method) contained 119 mM NaCl, 5 mM NaHCO_3_, 5.4 mM KCl, 0.35 mM Na_2_HPO_4_, 0.81 mM MgSO_4_, 0.44 mM KH_2_PO_4_ and 1.25 mM CaCl_2_ as a basic solution for perfusion. The perfusate was gassed with O_2_/CO_2_ (99:1, v/v) and its pH adjusted to 7.5. Livers were perfused at a flow rate of 4-5 ml/g liver/min and at a temperature of 30 °C. For determining the rates of gluconeogenic efflux from the perfused liver of both treated and control fish, livers were initially perfused for 30 min with isotonic medium, followed by infusion of gluconeogenic substrates (lactate, pyruvate or glutamate) separately in three sets of perfusion experiments each at a concentration of 5 mM (a concentration suitable for studying gluconeogenic efflux, Goswami et al. [17]) for 30 min. Effluents were collected at 2 min intervals for the determination of glucose efflux from the perfused liver and the steady-state efflux of glucose, obtained between 22 to 30 min of infusion of substrates, was used to calculate the rates of gluconeogenic fluxes. A steady state continuous efflux of glucose normally occurs from the perfused liver while perfusing with isotonic medium at least for 100-120 min (results not shown). Therefore, the rates of gluconeogenic fluxes were calculated by subtracting the value of steady-state efflux of glucose, obtained just before infusion, from the value of steady state efflux obtained after 20 min of infusion of gluconeogenic substrates [17].

### Estimation

For estimation of glucose in the perfusate, 10 µl of 2 M perchloric acid (PCA) was added to 1 ml of effluent collected at 2 min intervals, and the precipitated protein was removed by centrifugation. The supernatant was neutralized by adding 10 µl of 2M NaOH before estimation of glucose. Concentrations of glucose in effluents were measured enzymatically following the method of Bergmeyer et al. [35]. 

### Enzyme assay

A 10% homogenate (w/v) of each frozen tissue was prepared in a homogenizing buffer containing 50 mM Tris-HCl buffer (pH 7.4), 0.25 M sucrose, 1 mM ethylene diamine tetra-acetic acid (EDTA), 2 mM MgCl_2_, 1 mM dithiothreitol (DTT), 3 mM 2-mercaptoethanol and a cocktail of protease inhibitor (Roche, Germany) using a motor driven Potter-Elvehjem type glass homogenizer with a Teflon pestle. The homogenate was treated with 0.5% Triton X-100 in 1:1 ratio for 30 min, followed by mild sonication for 30×2 s. The homogenate was then centrifuged at 10,000 × g for 10 min and the supernatant was used for assaying the enzymes. All steps were carried out at 4°C.

The phosphoenolpyruvate carboxykinase (PEPCK) was assayed following the method of Mommsen et al. [36] with two-step enzymatic reactions. Fructose 1, 6-biphosphatase (FBPase) was assayed following the method of Mommsen et al. [36] with three step enzymatic reactions. Glucose-6-phosphatase (G6Pase) was assayed following the method of Nordlie and Arion [37]. In case of G6Pase, the reaction was stopped by the addition of 0.5 ml 10% perchloric acid after a specific period of time and the inorganic phosphate formed was estimated in the supernatant spectrophotometrically at 700 nm following Fiske and Subbarow [38] against a tissue blank, and expressed as enzyme activity. The decrease in absorbance (due to oxidation of NADH to NAD^+^) in case of PEPCK, the increase in absorbance (due to reduction of NADP^+^ to NADPH) in case of FBPase were recorded at 30 s interval at 340 nm in a UV-visible spectrophotometer (Varian, Model Cary 50) fitted with a peltier temperature-controlled device. One unit of enzyme activity was expressed as that amount of enzyme which catalyzed the oxidation of 1 µmol of NADH h^-1^ for PEPCK, or the reduction of 1 µmol of NADP^+^h^-1^at 30°C. For G6Pase, one unit of enzyme activity was expressed as that amount which catalyzed the formation of 1 µmol of inorganic phosphate h^-1^ at 30°C.

### Western blot

Western blot analyses of different gluconeogenic enzymes such as PEPCK, FBPase and G6Pase in different tissues of singhi catfish were performed following standard methods, the details of which were described in Saha et al. [39]. 

### RNA extraction and cDNA synthesis

The total RNA was isolated from liver and kidney tissues using TRI^®^ Reagent (Sigma Chemicals, St. Louis, USA), following Rio et al. [40]. The RNA solution was then further purified using the RNAase miniprotocol for RNA cleanup (Qiagen, Germany). Purified RNA was quantified spectrophotometrically, diluted to 5 µg/µl and electrophoresed on 1% agarose gel stained with ethidium bromide to verify integrity. First strand cDNA was synthesized from 1 µg total RNA (DNase I-treated, Invitrogen) in a total volume of 20 µl with High Capacity cDNA Reverse Transcriptase kit (Applied Biosystems, USA) as per the standard protocol.

### Quantitative Real-Time PCR (qPCR)

The qPCR was performed in the 7500 FAST RT-PCR (Applied Biosystems, USA) with Power SYBR® Green PCR Master Mix (Applied Biosytems, USA). The reaction mixture of 25 µl each contained 12.5 µl of 2x SYBR Green/ROX PCR Master Mix (Applied Biosystems, USA), 2.5 µl of cDNA, 8 pmoles of each primer and 6 µl of MilliQ H_2_O. The PCR conditions were 50 °C for 2 min, 95 °C for 10 min, followed by 40 cycles of 95 °C for 15 s and 54 °C 1 min for PECK, 57 °C 1 min for FBPase and 55 °C 1 min for G6Pase. Data were collected at 54 °C, 57 °C and 55 °C for PEPCK, FBPase and G6Pase, respectively. The qPCR was performed in triplicate and negative controls using no cDNA were run for each gene. Melting curve analysis was used to re-confirm amplification of only a single PCR product. The level of β-actin was invariant between the control and treated fish validating its choice as an endogenous control. Fold changes of PEPCK, FBPase and G6Pase genes in treated fish compared to untreated controls were calculated using the modified delta-delta C_T_ method [41,42].

The primer pairs were chosen from the published cDNA sequences of *Heteropneustes fossilis* PEPCK (FJ594279), FBPase (GQ860954), G6Pase (GU131155) and β-actin (FJ409641). The primers for PEPCK were: forward (5′-CGG GAA CCT CAC TGA AGA CAA-3′) and reverse (5′-GTG AAT ATC GTG TTC TTT GAA-3′), for FBPase forward (5′-GCA GCG CCA CCA TGA TAG T-3′) and reverse (5′-TCC AGC ATG AAG CAG TTG ACA-3′), for G6Pase forward (5′-TGA AGG CTG TGG GTG TGGAT-3′) and reverse (5′-ACG CAC CAT GTC TGA GCT TTT-3′), and for β-actin the primers were: forward (5'-CG TGA CAT CAA GGA GAA GCT-3') and reverse (5'-TGC CCA TCT CCT GCT CAA AG-3'), which were designed with the help of Primer Express Software 3.0 (Applied Biosystems, USA).

### Immunocytochemistry

Liver and kidney of both control and treated fish were excised and processed for immunostaining following Choudhury and Saha [43]. The PEPCK and G6Pase antibody rose in goat and FBPase antibody rose in rabbit (1:20) were applied for 2 h in a wet chamber at room temperature. After washing with PBS, the slides were incubated for 2 h in Cy3-conjugated rabbit anti-goat IgG for PEPCK and G6Pase and Cy3-conjugated goat anti-rabbit IgG for FBPase (1:500) in a dark wet chamber. After final washing, the sections were covered with Vectashield mounting medium with DAPI (Vector Laboratories, USA). Another set of slides were processed in the same way except incubation with primary antibodies, which served as negative controls. Immunostained sections were analyzed in a confocal laser microscope (Leica, TCS SP5, Germany). Cross-talk of fluorochromes was excluded by the use of the acousto optical tunable filter. The entire depth of a section was scanned in 1 µm steps. The resulting stacks of pictures were mounted as single projections.

### Chemicals

Enzymes, co-enzymes, substrates and oligonucleotide primers were purchased from Sigma Chemicals (St. Louis, USA). The PEPCK, G6Pase goat and FBPase rabbit polyclonal antibodies were purchased from Santa Cruz Biotechnology (USA). Other chemicals were of analytical grades and were obtained from local sources. MilliQ water was used in all preparations.

### Statistical analysis

The data collected from different replicates, were statistically analyzed and presented as mean ± S.E.M (*n* = number of animals in each set of experiment). Student’s *t*-test followed by multiple comparisons of means by Student-Newman-Keuls multiple range test were performed to evaluate differences between means where applicable. Differences with *P*<0.05 were regarded as statistically significant.

## Results

### Effect of environmental hypertonicity on blood osmolarity and tissue water content

In situ exposure of singhi catfish in hypertonic environment (300 mOsmol.l^-1^) led to a significant (*P*<0.05) increase of blood osmolarity from 265 ± 4 to 320 ± 5 mOsmol.l^-1^ (21%) after 7 days and to 332 ± 6 mOsmol.l^-1^ (25%) after 14 days (Table 1). This also led to decreases of water content in liver, and kidney tissues by 11.2 and 9.5%, respectively, after 7 days with no further changes at later stages of exposure (Table 2).

**Table 1:** Effect of environmental hypertonicity (300 mOsmol.l^-1^) on plasma osmolarity of singhi catfish.

**Blood osmolarity (mOsmol.l^-1^)**		
**Control**	**7 days treated**	**14 days treated**
265±2	318±5**^*a*^**	330±4**^*b*^**

^a,b^: Significantly different at *P*<0.05 and <0.01 levels, respectively, compared to control value (Student’s *t*-test).

Values are expressed as mean ± SEM (n=5).

**Table 2:** Effect of environmental hypertonicity (300 mOsmol.l^-1^) on water content in liver and kidney tissues of singhi catfish.

**Tissue**	**% decrease of water content**	
	**7 days treated**	**14 days treated**
Liver	-11.2±1.2**^*a*^**	-11.3±1.1**^*a*^**
Kidney	-9.5±0.7**^*a*^**	-9.7±0.8**^*a*^**

^a^ : Significantly different at *P*<0.05 level compared to control values (Student’s *t*-test).

Values are expressed as mean ± SEM (N=5).

### Effect of environmental hypertonicity on gluconeogenic fluxes from the perfused liver

**Figure 1:**
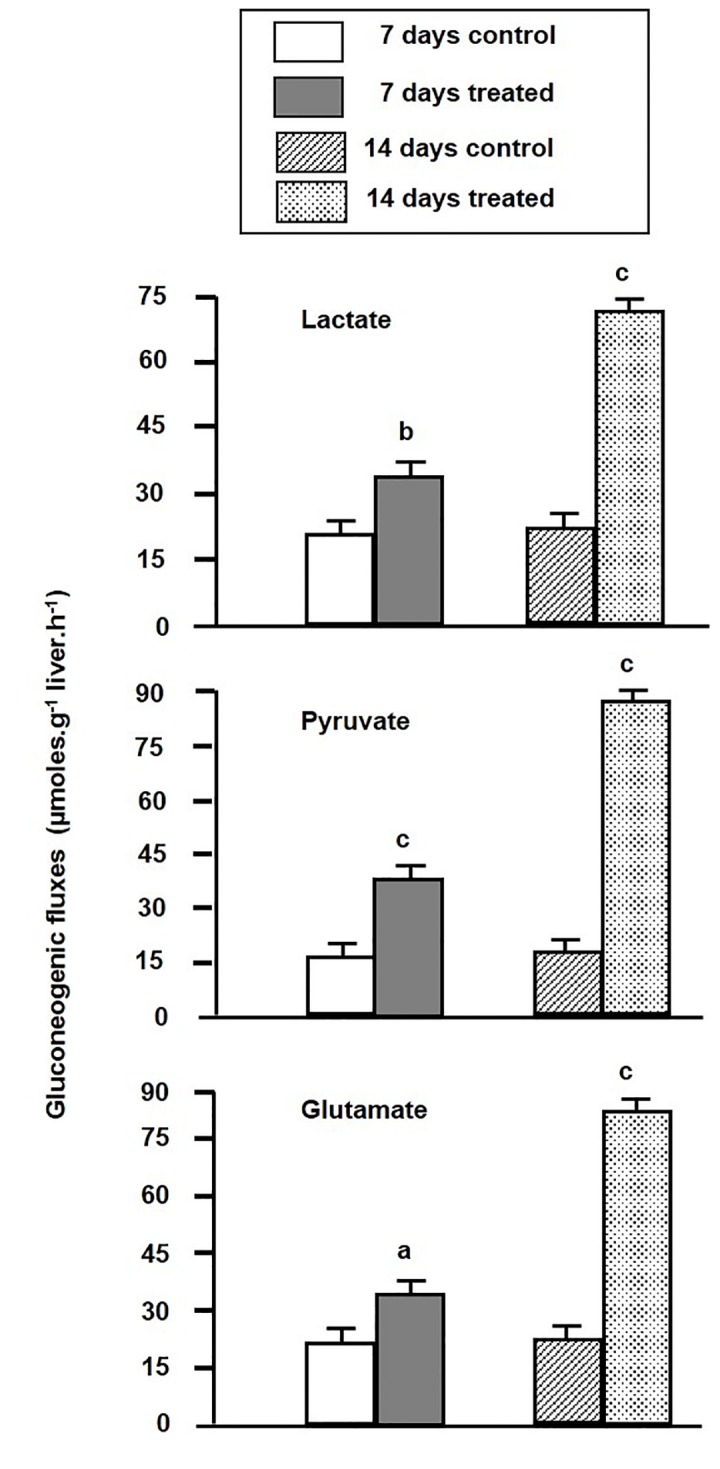
Gluconeogenic fluxes from the perfused liver. The changes of gluconeogenic fluxes (µmoles.g^-1^ liver.h^-1^) from the perfused liver of singhi catfish were measured both in control and in fish exposed to hypertonic environment for different time intervals. Values are plotted as mean ± S.E.M (n = 5). Livers of both control and hypertonically-treated fish were perfused with isotonic medium for 30 min, followed by infusion of gluconeogenic substrates (5 mM) for 30 min, and then again without the substrate for 20 min. The steady state fluxes of glucose between 22-30 min of perfusion and between 52-60 min of perfusion were used to calculate the rate of gluconeogenic fluxes in presence of different gluconeogenic substrates (mentioned in details in materials and methods section).

Effect of environmental hypertonicity on gluconeogenic fluxes from the liver organ of singhi catfish, as a measure of gluconeogenic activity, was studied by the perfusion technique in presence of three different potential gluconeogenic substrates separately such as lactate, pyruvate and glutamate (Figure 1). In control fish, the maximum gluconeogenic efflux from the perfused liver was recorded in presence of glutamate (22.2 ± 0.08 µmoles.g^-1^ liver.h^-1^), followed by the presence of lactate (20.4 ± 0.12 µmoles.g^-1^ liver.h^-1^) and pyruvate (15.6 ± 0.12 µmoles.g^-1^ liver.h^-1^). Interestingly, the gluconeogenic fluxes from the perfused liver of fish exposed to hypertonic environment increased significantly by 1.61, 2.38 and 1.51 fold, respectively, in presence of lactate, pyruvate and glutamate after 7 days, which further rose to 3.30, 5.13 and 3.44 fold after 14 days.

### Effect of environmental hypertonicity on activities of gluconeogenic enzymes

**Figure 2:**
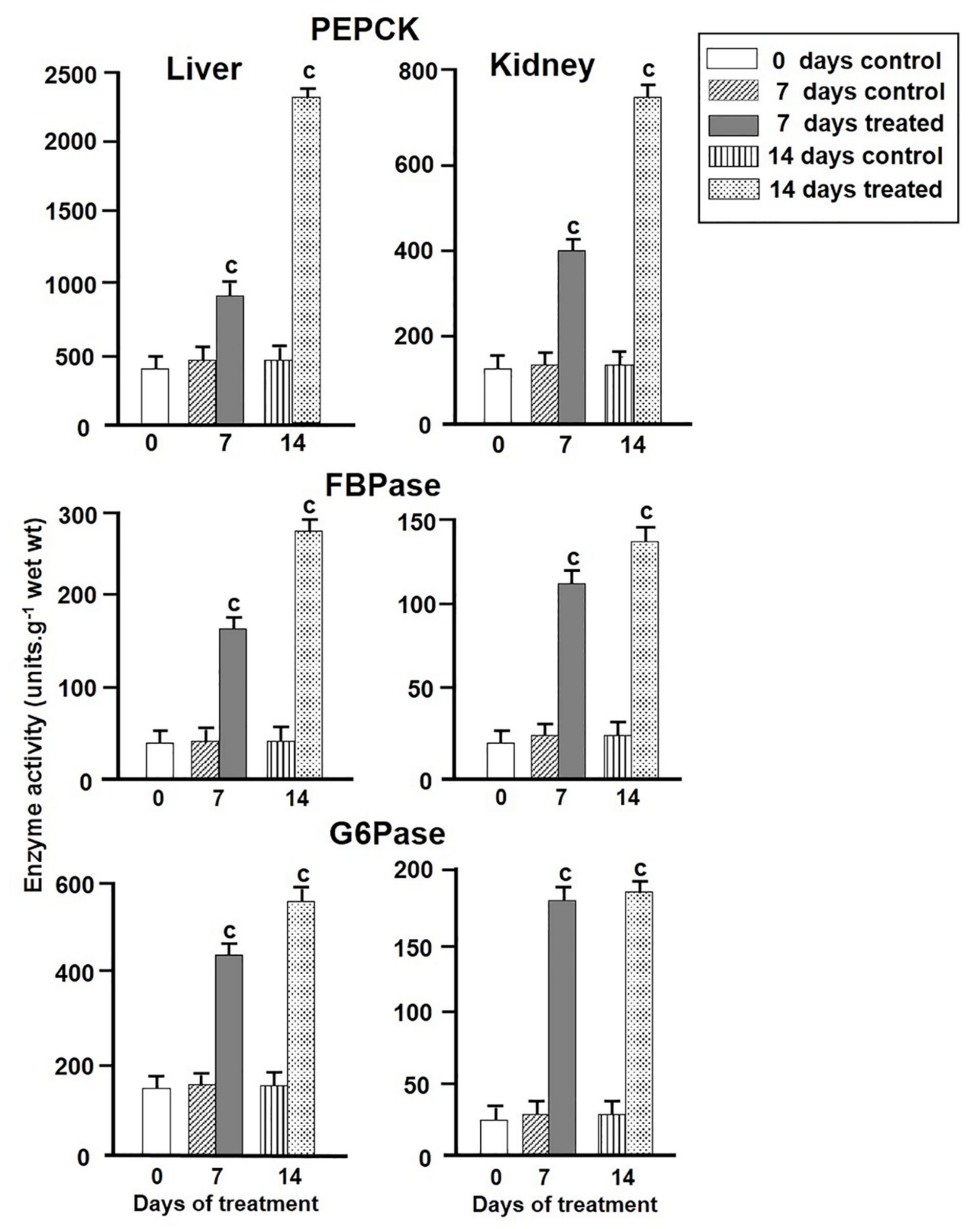
The activity of gluconeogenic enzymes. Changes in activities (units.g^-1^ wet wt) of different gluconeogenic enzymes in singhi catfish were analysed both in control and in fish exposed to hypertonic environment for different time intervals. Values are plotted as mean ± S.E.M (n = 5). One unit of enzyme activity was expressed as that amount of enzyme that catalyzed the oxidation of 1 µmol of NADH h^-1^ at 30 °C in case of PEPCK, reduction of 1 µmol of NADP^+^ h^-1^ at 30 °C in case of FBPase and 1 µmol of inorganic phosphate formed h^-1^ at 30 °C in case of G6Pase. ^c^ :P value significant at <0.001 level compared to respective controls (Student’s *t*-test).

In control fish, significant levels of activities of three key gluconeogenic enzymes namely PEPCK, FBPase and G6Pase were detected both in liver and kidney tissues (two key gluconeogenic tissues) of singhi catfish, which further enhanced significantly in fish exposed to hypertonic environment (Figure 2). In liver, the activities of PEPCK, FBPase and G6Pase increased significantly by 2.00, 2.89 and 3.84 fold, respectively, after 7 days, followed by further increase by 4.88, 3.57 and 6.16 fold after 14 days of exposure. In kidney, the activities of PEPCK, FBPase and G6Pase increased significantly by 2.92, 6.05 and 4.47 fold, respectively, after 7 days, which increased further by 4.66, 6.09 and 5.25 fold after 14 days of exposure.

### Effect of environmental hypertonicity on the abundance of gluconeogenic enzyme proteins

**Figure 3:**
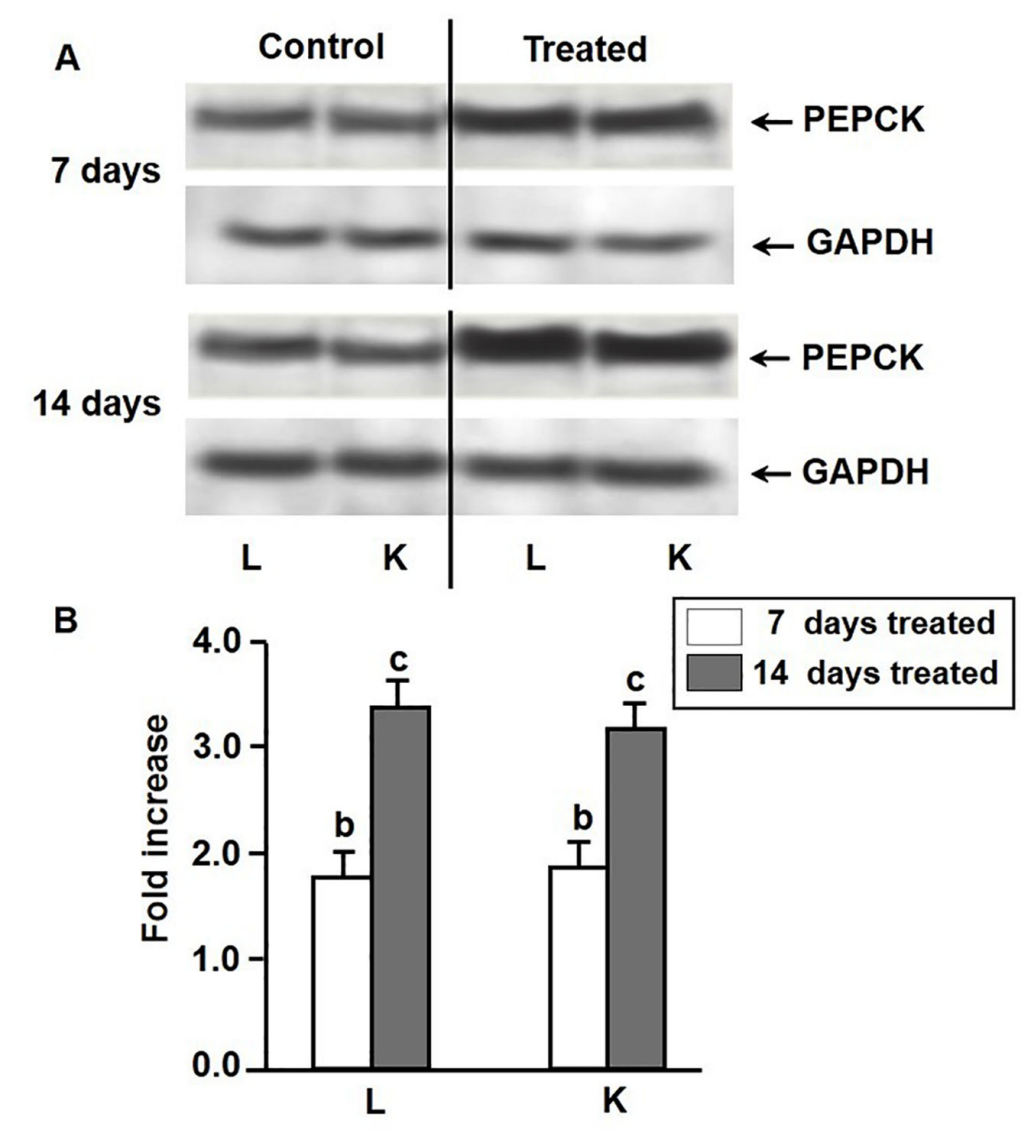
Expression pattern of PEPCK enzyme protein. Western blot analysis showing changes in the levels of expression of PEPCK enzyme protein in liver (L) and kidney (K) of singhi catfish following exposure to environmental hypertonicity at different time intervals. (A) A representative plot of 5 individual experiments. GAPDH was taken as a protein loading control. (B) Densitometric analysis showing the fold increase of PEPCK protein concentration in treated fish compared to respective controls. Values are plotted as mean ± S.E.M. (n = 5). ^c^ :P value significant at <0.001 level compared to respective controls (Student’s *t*-test).

**Figure 4:**
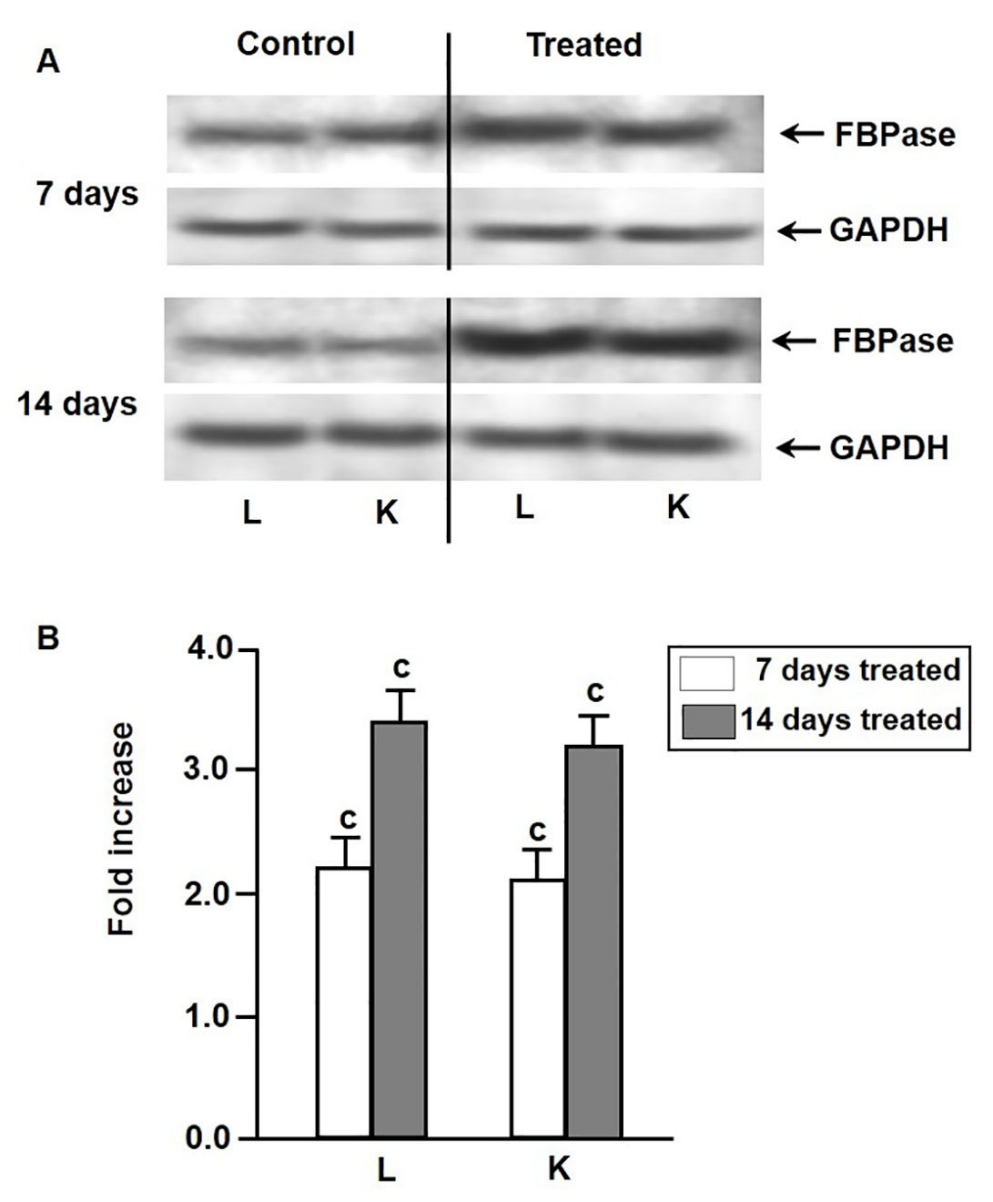
Expression pattern of FBPase enzyme protein. Western blot analysis showing changes in the levels of expression of FBPase enzyme protein in liver (L) and kidney (K) of singhi catfish following exposure to environmental hypertonicity at different time intervals. (A) A representative plot of 5 individual experiments. GAPDH was taken as a protein loading control. (B) Densitometric analysis showing the fold increase of FBPase protein concentration in treated fish compared to respective controls. Values are plotted as mean ± S.E.M. (n = 5). ^c^ :P value significant at <0.001 level compared to respective controls (Student’s *t*-test).

**Figure 5:**
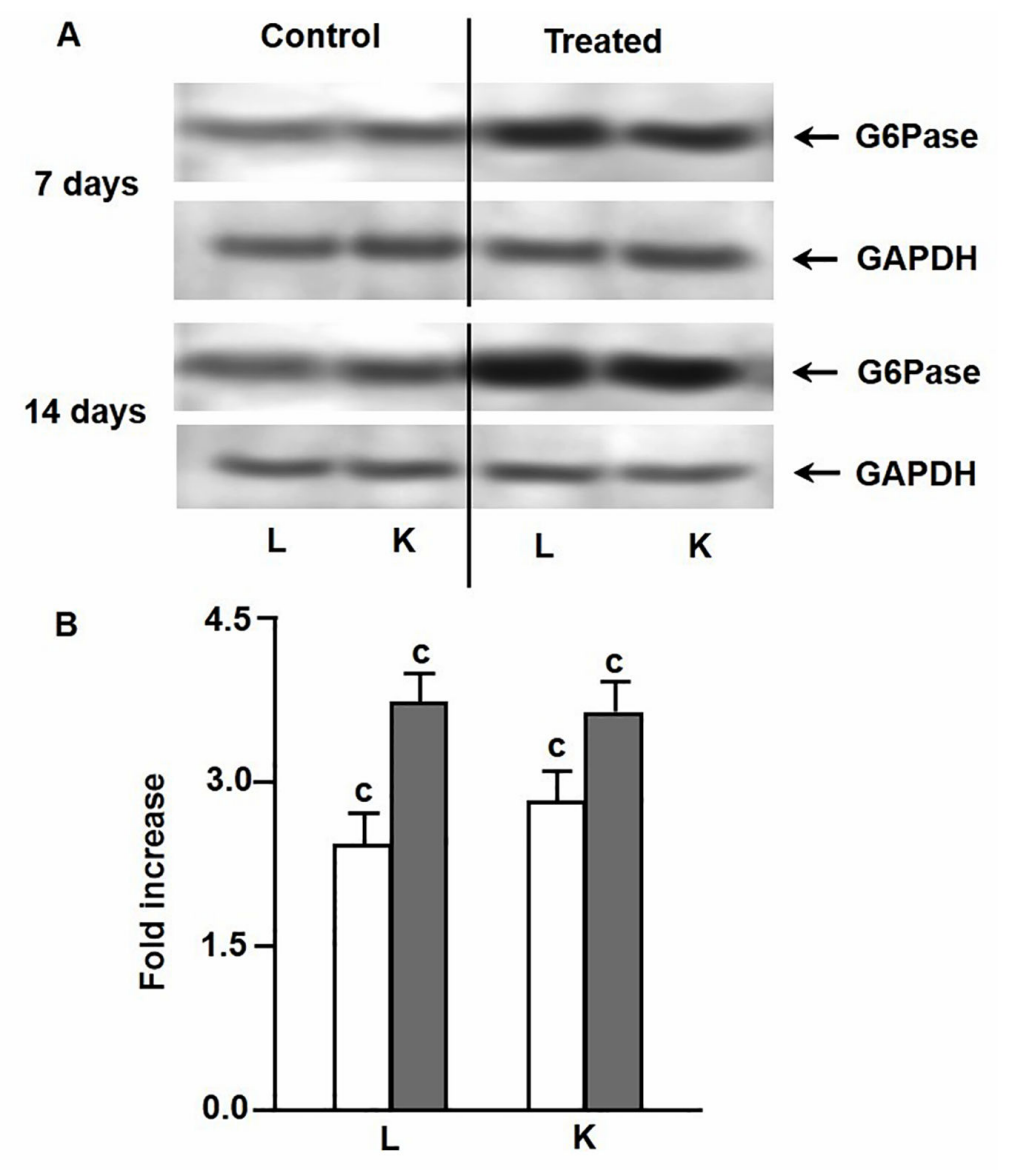
Expression pattern of G6Pase enzyme protein. Western blot analysis showing changes in the levels of expression of G6Pase enzyme protein in liver (L) and kidney (K) of singhi catfish following exposure to environmental hypertonicity at different time intervals. (A) A representative plot of 5 individual experiments. GAPDH was taken as a protein loading control. (B) Densitometric analysis showing the fold increase of G6Pase protein concentration in treated fish compared to respective controls. Values are plotted as mean ± S.E.M. (n = 5). ^c^ :P value significant at <0.001 level compared to respective controls (Student’s *t*-test).

As evidenced by Western blot analysis, the increases of activities of PEPCK and FBPase and G6Pase in liver and kidney tissues of singhi catfish during exposure to environmental hypertonicity was accompanied by a significant increase in the abundance of these enzyme proteins in both the tissues (Figures 3-5). In case of PEPCK, the enzyme protein concentration increased by 1.8 and 1.9 fold in liver and kidney, respectively, after 7 days, with a further increase by 3.4 and 3.2 fold after 14 days of exposure (Figure 3). In case of FBPase, it increased by 2.2 and 2.1 fold in liver and kidney tissues, respectively, after 7 days of exposure, which further rose to 3.4 and 3.2 fold after 14 days (Figure 4). Similarly, the abundance of G6Pase enzyme protein also increased by 2.4 and 2.8 fold after 7 days of exposure, followed by further increase by 3.7 and 3.6 fold after 14 days of exposure in liver and kidney tissues, respectively (Figure 5).

### Effect of environmental hypertonicity on the expression of mRNAs for gluconeogenic enzymes

**Figure 6:**
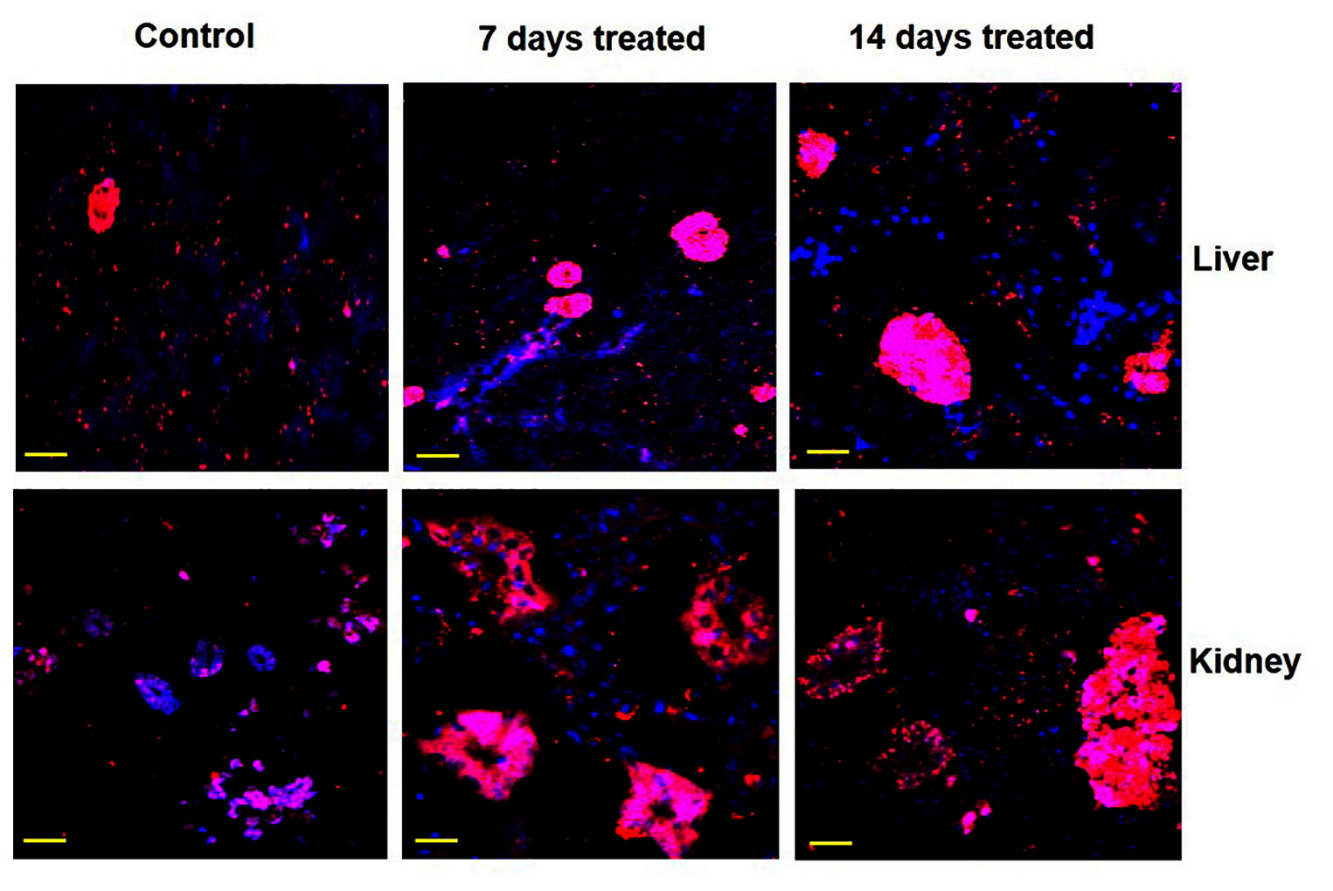
Zonal localization of PEPCK. Immunocytochemical analysis showing the localized expression of PEPCK in liver and kidney tissues of singhi catfish following exposure to hypertonic environment at different time intervals. Representative pictures of three independent experiments are shown. Nucleus – blue (DAPI); PEPCK – red (cy3). Scale bar: 55 µm.

Real-time qPCR analysis on the expression of different mRNAs of three gluconeogenic enzymes indicated that the abundance mRNAs for all the enzymes got significantly elevated both in liver and kidney tissues following exposure to hypertonic environment (Figure 6). In case of PEPCK, the mRNA level increased significantly by 2.5 and 3.6 fold in liver and kidney, respectively, after 7 days, which further rose to 4.7 and 5.2 fold after 14 days of exposure. Similarly, in case of FBPase, the mRNA level increased by 2.7 and 2.2 fold in liver and kidney tissues, respectively, after 7 days, followed by further increase by 3.5 and 4.7 fold after 14 days of exposure. The level of mRNA for G6Pase also increased significantly by 2.2 and 3.1 fold, respectively, in liver and kidney tissues after 7 days, which further rose to 3.4 and 4.6 fold after 14 days of exposure to environmental hypertonicity. 

### Immunolocalization of gluconeogenic enzymes under environmental hypertonicity

**Figure 7:**
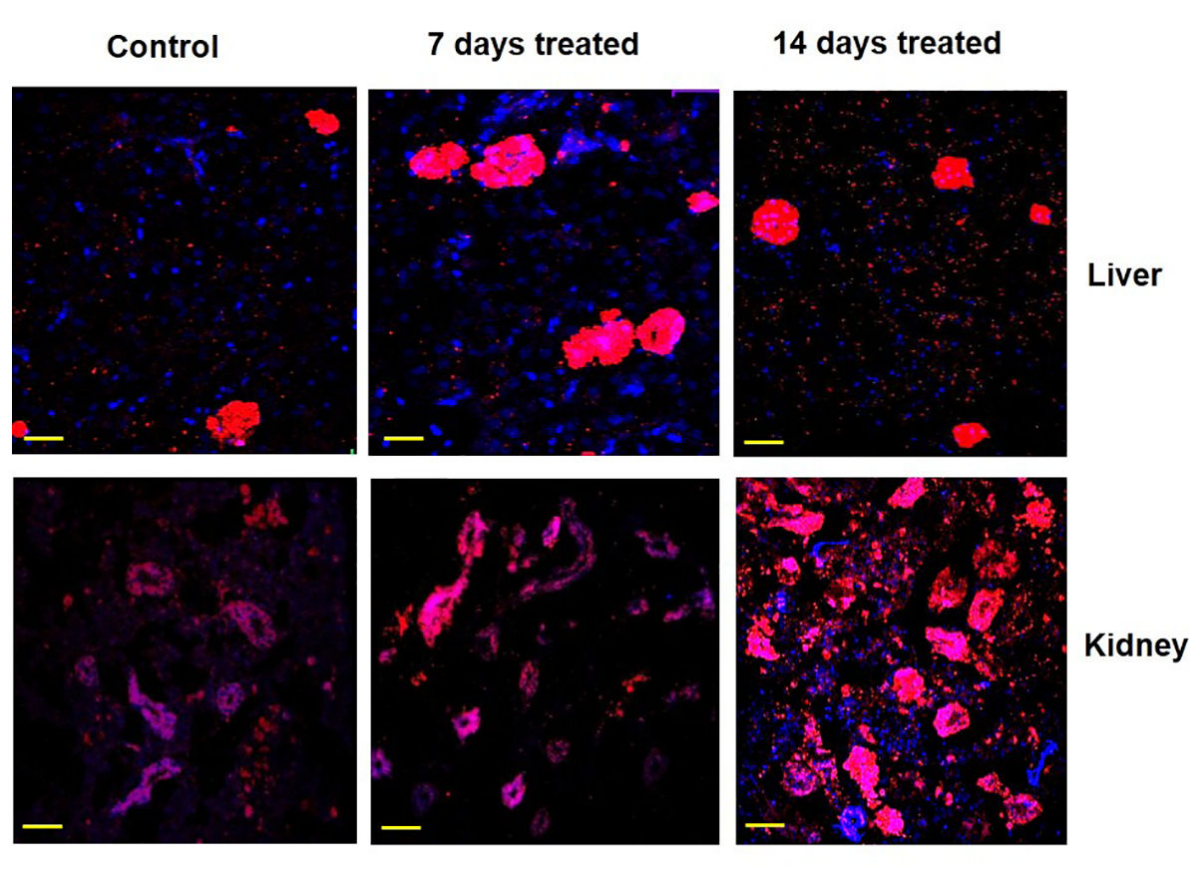
Zonal localization of FBPase. Immunocytochemical analysis showing the localized expression of FBPase in liver and kidney tissues of singhi catfish following exposure to hypertonic environment at different time intervals. Representative pictures of three independent experiments are shown. Nucleus – blue (DAPI); FBPase – red (cy3). Scale bar: 55 µm.

**Figure 8:**
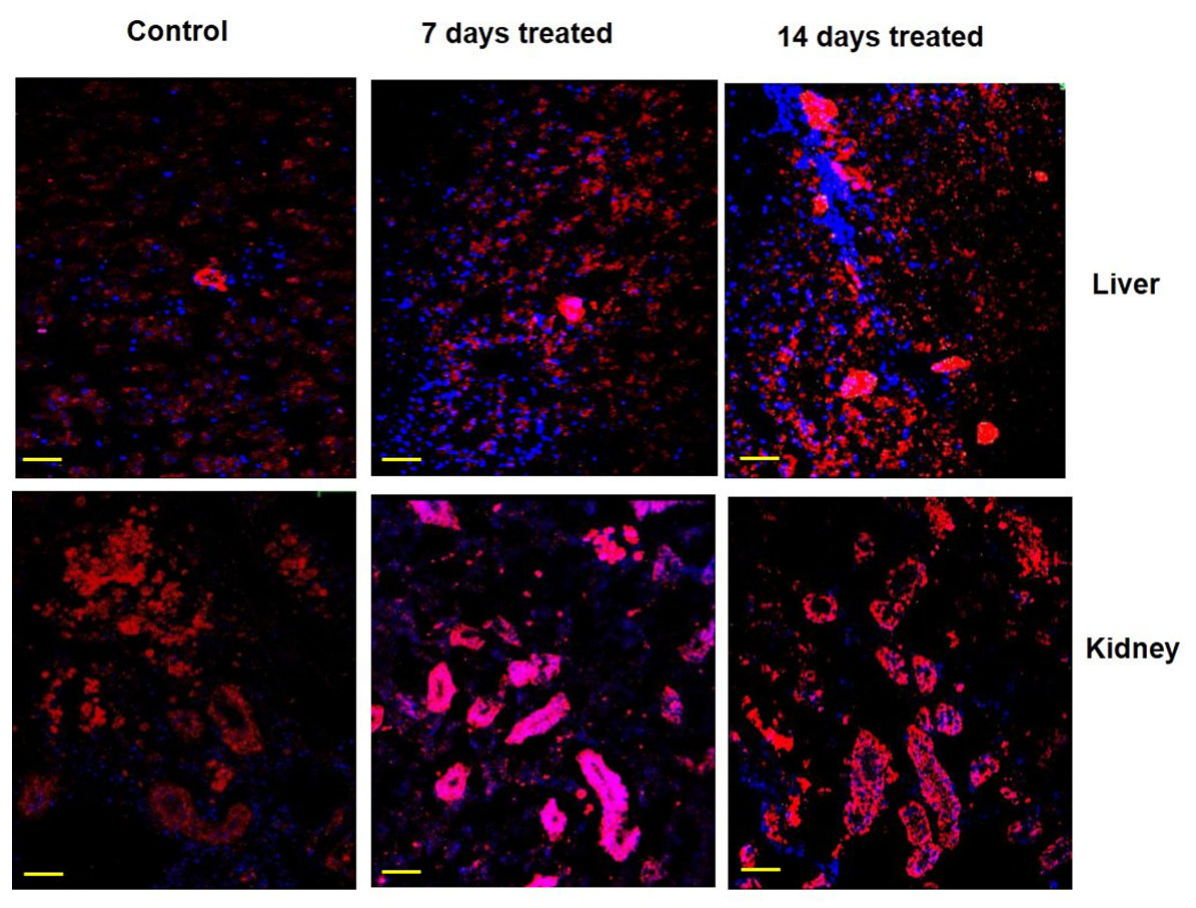
Zonal localization of G6Pase. Immunocytochemical analysis showing the localized expression of G6Pase in liver and kidney tissues of singhi catfish following exposure to hypertonic environment for different time intervals. Representative pictures of three independent experiments are shown. Nucleus – blue (DAPI); G6Pase – red (cy3). Scale bar: 55 µm.

**Figure 9:**
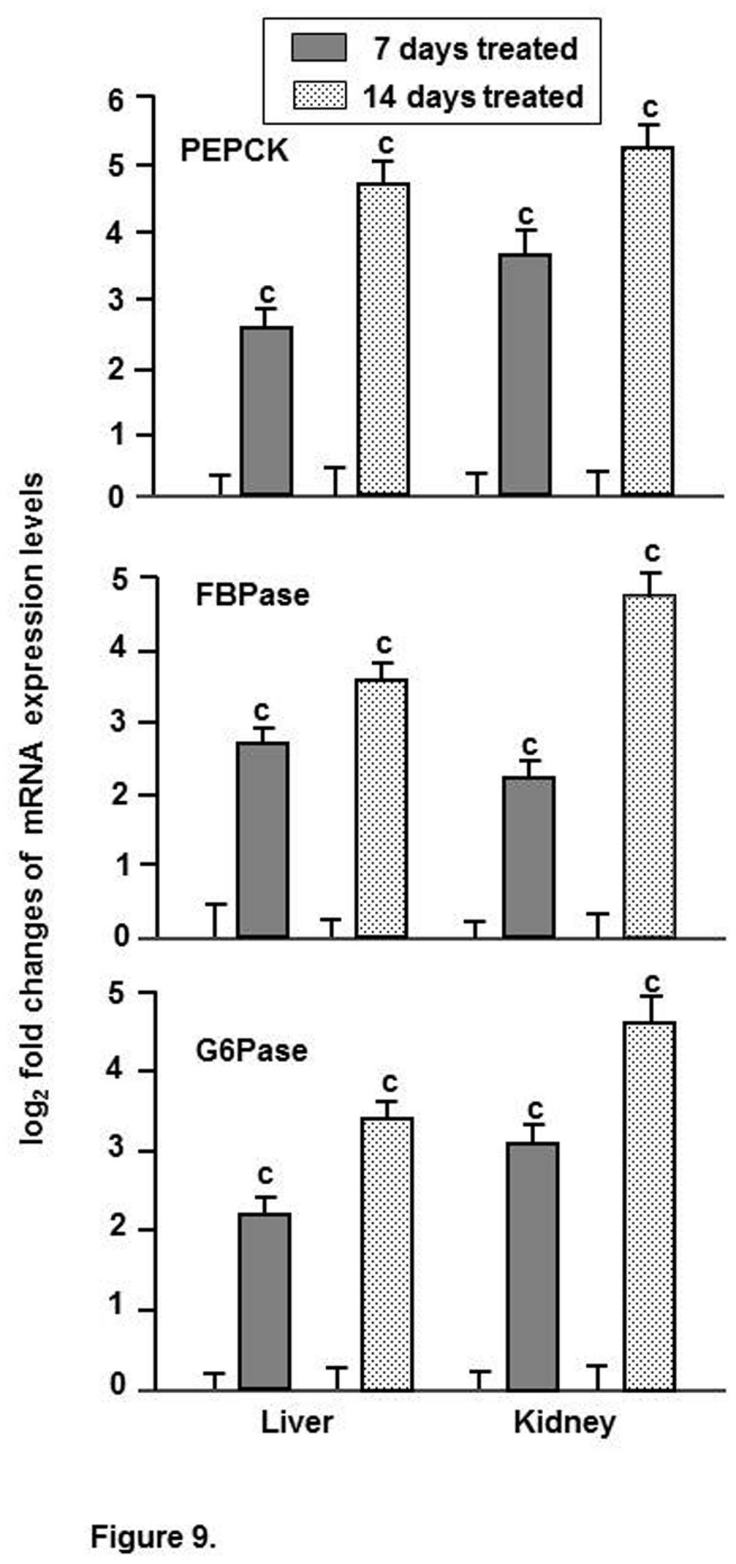
Expression of mRNAs for gluconeogenic enzymes. qPCR analysis showing the levels of relative expression of mRNAs for different gluconeogenic enzymes in liver and kidney tissues of singhi catfish following exposure to environmental hypertonicity at different time intervals. Values are plotted as mean ± S.E.M. (n = 5) ^c^ : *P* value significant at <0.001 level, compared to respective controls (Student’s *t*-test)

The expression pattern and zonal localization of PEPCK, FBPase and G6Pase enzymes were observed by immunocytochemical analysis under confocal laser scanning microscope in two main gluconeogenic tissues (liver and kidney) of control and also in fish after exposure to hypertonic environment by using a monoclonal antibodies specific to PEPCK, FBPase and G6Pase (Figures 7-9). Labeling specificity was confirmed by the absence of signal in parallel control sections treated without the primary antibody (data not shown). In the liver of control fish, the signals for these gluconeogenic enzymes were mainly localized in the cluster of hepatic sinusoidal endothelial cells. After exposing the fish in hypertonic environment, the signals became more intense, but in the same localized places. In the kidney of control fish, the signals for these gluconeogenic enzymes were mainly localized in the proximal and distal tubules in the cortex region with further enhancement of signals after exposing the fish in hypertonic environment.

## Discussion

Reports on the influences of various environmental factors such as temperature, hypoxia, starvation, and certain hormones on carbohydrate metabolism including gluconeogenesis in different fish species are well documented by several workers (for review, see 14). There are also reports on the influence of dietary carbohydrates on gluconeogenesis in trout, carp and sea bream [15,44,45]. However, reports on the influence of environmental hypertonicity on gluconeogenic activity in teleosts are scanty. More recently, it has been demonstrated that the alterations of hepatic cell volume due to anisotonicity lead to changes in carbohydrate and oxidative metabolisms in the perfused liver of air-breathing walking catfish [16,17,29], and also the autophagic proteolysis [25] and the rates of protein synthesis in isolated hepatocytes of the walking catfish [46].

The present work clearly demonstrated that the gluconeogenic activity and expression of different gluconeogenic enzyme genes viz. PEPCK, FBPase and G6Pase could be stimulated by environmental hypertonicity in singhi catfish by exposing the fish in situ in 300 mM mannitol (equivalent to 300 mOsmol.l^-1^osmolarity). As a consequence, the gluconeogenic fluxes from the perfused liver of fish exposed to hypertonic environment with all the three substrates (lactate, pyruvate and glutamate), which are considered to be most potential gluconeogenic substrates at least in another closely related species of air-breathing catfish (*C. batrachus*) [17], got significantly elevated. The maximum elevation was seen with lactate and pyruvate, indicating that an active Cori and alanine cycle is prevailing in this singhi catfish. Thus, lactate and pyruvate gluconeogenesis could be one of the major sources of energy in this catfish under various environmental constraints including that of hypertonicity. Further, this catfish is predominantly carnivorous in its feeding habit, and primary depends on high protein and low carbohydrate diets [47]. Fishes are known to use lactate as an energy substrate during acclimation to hypertonic stress as evidenced from the previous studies of changes in plasma lactate levels, as well as lactate content and lactate dehydrogenase expression/activity in osmoregulatory organs [48-52]. Amino acid gluconeogenesis, which has great physiological significance, was reported in walking catfish and also in trout [17,53]. A sufficient and timely energy supply is a prerequisite for the operation of iono- and osmoregulatory mechanisms in fish. Carbohydrate metabolism appears to play a major role in the energy supply for iono- and osmoregulation, and liver is known to be the major source supplying carbohydrate metabolites to osmoregulatory organsduring acclimation to hypertonic stress. Many genes associated with many metabolic processes such as electron transport chain, TCA cycle, glycolysis, polysaccharide metabolism, fatty acid catabolism, peptide cleavage and proteolysis are reported to be up-regulated in different fish species under hypertonic stress [52]. Hypertonicity is also reported to stimulate the autophagic proteolysis in walking catfish liver [25]. Therefore, stimulation of proteolysis in response to hypertonicity should favour gluconeogenesis from proteolysis-derived amino acids as a coordination of a functionally linked physiological process in response to changes of cell volume under hypertonic stress. 

In this study, parallel to induction of gluconeogenesis, increases in the activity of key gluconeogenic enzymes by 2-6 fold, accompanied by increases in the abundance of enzyme proteinsby about 2-4 fold and mRNAs by about 2-5 for in liver and kidney tissues of fish exposed to hypertonic environment were observed. Thus, the induction of PEPCK, FBPase and G6Pase activities appeared to be mainly associated with transcriptional regulation of genes of these enzymes under hypertonic stress.

The enzyme PEPCK is known to occur in two isoforms (the mitochondrial and the cytosolic forms) with different distribution and regulatory patterns in various groups of vertebrates [54]. A full length PEPCK cDNA coding for mitochondrial isoform has been cloned in rainbow trout liver [44]. It has been demonstrated that in animals in which both the mitochondrial and the cytosolic forms occur such as in chicken [55], only the cytosolic form is acutely regulated by diet and hormones, whereas the gene for mitochondrial PEPCK is largely constitutive in its pattern of expression [54]. Similarly, in rainbow trout, the PEPCK gene, which is exclusively codes for the mitochondrial type of PEPCK, could not be regulated by dietary carbohydrates [56]. But, with our present data and with partial sequence data of PEPCK (FJ594279), also for FBPase (GQ86094) and G6Pase (GU131155) genes from this singhi catfish, which could not discriminate between cytosolic and mitochondrial isoforms, it may be difficult to conclude about which isoforms were regulated at the transcriptional level resulting to an increase of activity of these enzymes in this singhi catfish during hypertonicity. However, compartmentalization of gluconeogenic enzymes could be of regulatory significance in this catfish as suggested in other fish species such as plaice (*Pluronectis platessa*) [57] and in chicken [55]. Upregulation of PEPCK and FBPase genes at transcriptional level has been demonstrated in perfused rat liver and in H4IIE rat hepatoma cells within 3-6 h of hypertonic exposure and correlated with the hydration status of hepatic cells [58,59]. In situ exposure of singhi catfish in hypertonic environment led to a significant increase of blood osmolarity, which was accompanied by a decrease of water content both in liver and kidney tissues. In walking catfish, the hepatocytes were reported to remain partly swollen/shrunken state in hypo-/hypertonic conditional though it possesses a very efficient volume regulatory mechanisms, shown both in intact liver organ [23,25] and also in isolated cells [46]. The same is also probably true in case of singhi catfish, since the water content both in liver and kidney tissues decreased significantly during exposure to hypertonic environment. Therefore, the induction of activities of PEPCK, FBPase and G6Pase along with more abundance of enzyme proteins and mRNAs, following in situ exposure to hypertonic environment, could also be as a result of decreasing water content or cell volume in both the gluconeogenic tissues of this catfish. Further, it was demonstrated that the increase of cell volume due to hypotonicity and decrease of cell volume due to hypertonicity cause decrease and increase of gluconeogenic activity, respectively, from different substrates using an intact liver organ of walking catfish under perfusion condition [17]. More recently, stimulation of gluconeogenesis during exposure to high saline environment (150 mM NaCl) has also been reported in the walking catfish [60]. Thus, the reports on the regulation of gluconeogenesis by changing the hydration status or alterations of cell volume in different gluconeogenic tissues add a new event to the complex regulation of PEPCK, FBPase and G6Pase genes of gluconeogenic enzymes in air-breathing catfish.

A major question arises now from this study concerning the mechanisms by which environmental hypertonicity, thereby decreasing the cellular hydration status of different tissues, exerts an effect on PEPCK, FBPase and G6Pase genes transcription and also enhances the gluconeogenic activity. Modulation of all the three mRNA levels due to hypertonicity appears to be due to up-regulation of gene transcription rather than mRNA stability, since increases in mRNA levels were also accompanied by more abundance of all the three enzyme proteins. In mammals, the PEPCK activity is generally altered by transcriptional regulation of expression of its gene [58]. Further, the PEPCK gene in mammals encoding the cytosolic isoform is known to be under nutritional and hormonal control, which is not the case for mitochondrial isoform and is known to be constitutively expressed independently of nutritional status of the animal, unfed versus fed with or without carbohydrate or fed with increased dietary proportion of protein levels [44,61-64]. As noticed in mammalian system during varied physiological stimuli, including dietary carbohydrate content, nutritional status, and various hormones [54,65], the transcription of PEPCK in singhi catfish may also be tightly controlled by various pre-existing transcription factors that bind to PEPCK promoter due to altered phosphorylation status in response to hypertonicity. In rainbow trout, insulin was found to inhibit the expression of PEPCK at the transcriptional level [66] through the activation of the protein kinase AKT [67]. In addition to transcriptional regulation of PEPCK, TIP60-dependent acylation of PEPCK, as a posttranslational modification, could be another means of induction of activity during exposure to environmental hypertonicity and other environmentally-related insults, as shown recently as a cause for increasing its activity in mammals during fasting [68]. In mammals, FBPase gene expression is regulated both by transcriptional and post transcriptional mechanisms [69]. In rainbow trout, expression of FBPase was suggested to be poorly regulated by feeding and re-feeding [56,63,70], whereas starvation was found to significantly increase the expression of FBPase gene in zebrafish [71]. Again in mammals, the hepatic expression of G6Pase is subjected to hormonal and nutritional regulation. Increasing of cAMP, due to starvation and hormones, was reported to stimulate G6Pase gene expression, whereas re-feeding and insulin both developed opposite effect [72,73]. Similarly, food deprivation was reported to increase hepatic expression of G6Pase in gilthead sea bream [61,74,75]. In case of singhi catfish, in addition to transcriptional regulation of gluconeogenic enzymes, there could be allosteric modulation of certain gluconeogenic enzymes under hypertonic stress to ensure a prompt adaptation to gluconeogenic fluxes leading to glucose homeostasis, and energy supply during ono- and osmoregulatory processes. However, to understand better about the possible mechanism(s) of regulation of gluconeogenesis during osmotic stress in this air-breathing catfish one requires to study further.

Immunocytochemical analysis clearly demonstrated the localized expression of gluconeogenic enzyme proteins in liver and kidney tissues and more expression of all the three gluconeogenic enzymes under hypertonic stress. In liver, the expression PEPCK, FBPase and G6Pase enzyme proteins were noticed in clusters of endothelial cells of sinusoids. This zonation of gluconeogenic enzymes and to remain in same localized place could as a consequence of predominance of gluconeogenesis over glycolysis as suggested by many workers in mammals [76-79]. In kidney of singhi catfish, all the three gluconeogenic enzymes were found to express mainly in proximal and distal tubular cells localized in the kidney cortex, indicating that the glucose synthesis is compartmentalized to the proximal tubule with more expression of all the three enzymes in the same localization after exposure to hypertonic environment. 

In conclusion, environmental hypertonicity leads to a stimulation of gluconeogenesis in the air-breathing singhi catfish by up-regulating the activities of key gluconeogenic enzymes as a consequence of transcriptional regulation of genes for gluconeogenic enzymes, since the induction of activities of gluconeogenic enzymes was accompanied by more abundance of key gluconeogenic enzyme proteins and mRNAs in liver and kidney tissues during exposure to hypertonic environment. Further, the gluconeogenic enzymes show localized expression in liver and kidney tissues with the possibility of more expression of these enzymes in same localized places. Furthermore, in addition to lactate and pyruvate gluconeogenesis, amino acid gluconeogenesis is also very much prevalent in this fish probably as a consequence of more abundance of amino acids due to induction of autophagic proteolysis during by hypertonic cell shrinkage shown in one related catfish (*C. batrachus*) [25]. These biochemical adaptational strategies, possibly as a consequence of changes of hydration status/cell volume of different cell types during environmental hypertonicity, would assist in maintaining glucose homeostasis and proper energy supply mainly to support metabolic demands for ion transport and other altered metabolic processes in this air-breathing singhi catfish.
